# Experimental Study of Failures of the Rigid Spinal Posterior Fixation System Under Compressive Load Conditions: A Cadaver Study

**DOI:** 10.7759/cureus.53961

**Published:** 2024-02-10

**Authors:** Takaya Kato, Tadashi Inaba, Sotaro Baba, Tadatsugu Morimoto, Tetsutaro Mizuno, Yuichi Kasai, Taweechok Wisanuyotin, Winai Sirichativapee, Weerachai Kosuwon, Permsak Paholpak

**Affiliations:** 1 Department of Social Innovation, Graduate School of Regional Innovation Studies, Mie University, Tsu, JPN; 2 Department of Mechanical Engineering, Graduate School of Engineering, Mie University, Tsu, JPN; 3 Department of Orthopaedic Surgery, Faculty of Medicine, Saga University, Saga, JPN; 4 Department of Orthopaedic Surgery, Seirei Hamamatsu General Hospital, Hamamatsu, JPN; 5 Department of Orthopaedics, Faculty of Medicine, Khon Kaen University, Khon Kaen, THA

**Keywords:** simulation models, instrumentation failure, strain, intradiscal pressure, biomechanics, spinal instrumentation, human cadaver experiment, lumbar spine

## Abstract

Background

Many studies have been conducted on the biomechanics of the spine to elucidate the fixation properties of spinal fusion surgery and the causes of instrumentation failure. Among these studies, there are some studies on load sharing in the spine and measurement using strain gauges and pressure gauges, but there is a lack of research on axial compressive loads.

Methods

Axial compressive load tests were performed on human cadaveric injured lumbar vertebrae fixed with pedicle screws (PS). Both the strain generated in the PS rod and the intradiscal pressure were measured. Subsequently, the stress generated in the PS rod and the load sharing of the spine and instrumentation were calculated.

Results

Even when only compressive load is applied, bending stress of more than 10 times the compression stress was generated in the rod, and the stress tended to concentrate on one rod. Rod deformation becomes kyphotic, in contrast to the lordotic deformation behavior of the lumbar spine. The stress shielding rate was approximately 40%, less than half.

Conclusions

This study obtained basic data useful for constructing and verifying numerical simulations that are effective for predicting and elucidating the causes of dislodgement and failure of spinal implants.

## Introduction

Many research studies have investigated spinal biomechanics [1-4]. Cripton et al. [5] created a model in which sequential injuries were made to lumbar functional spinal units stabilized with posterior instrumentation. Subsequently, these models were applied moment to investigate load sharing in the vertebral body and implant. The results showed that when anterior elements of the spine were severely injured, the majority of the stress generated was borne by the implant and that concentration of the stress could potentially occur on one of the rods. They indicated that this could be a cause of instrumentation failure. Cripton et al. [6] also reported that in vivo experiments may need to give sufficient consideration to the axial compressive load (initial load) produced even in the absence of accompanying movsequently, these models were appliedement and that the initial load affects the results of in vitro mechanical tests of the spine. However, that study did not investigate load sharing of the spine and implant at the time of implant fixation. Although studies on the loads on implants and load distribution between implants and the spine are scattered in previous research, biomechanical studies under simple compressive load conditions are not sufficient.

Therefore, as the most basic research area of spine biomechanics, we aimed to clarify the strain of the implant's rod and the load sharing between the spine and implants caused by simple axial compressive loads, thereby increasing our understanding of implant fixation and malfunctions. As common posterior fixation techniques for the lumbar spine, posterior lumbar fusion (PLF) and posterior lumbar interbody fusion (PLIF) are considered. In this study, as a preliminary step before considering anterior reconstruction, we focused on the most fundamental posterior reconstruction and created a model on human cadaver lumbar spines assuming injuries occur during PLF. We performed pedicle screws (PS) fixation on the model and measured the strain of the rod and the intradiscal pressure when axial compressive loads were applied. The results are expected to provide useful information for numerical analysis to predict the dislocation and fracture of implants, which have been rapidly developed in recent years.

This article includes portions of content posted to the Research Square preprint server on September 27, 2021.

## Materials and methods

Study design and setting

This research was approved by the Ethics Committee for Human Research at Khon Kaen University, Khon Kaen, Thailand (approval number: HE611293), and conducted in the Department of Orthopaedics at Khon Kaen University. Among the cadavers donated to the Department of Anatomy at Khon Kaen University during the research period, scoliosis, fractures, obvious degeneration, and osteoporosis were excluded, and specimens were prepared from the lumbar vertebrae of four cadavers. In addition, in the lumbar vertebrae, L5/S1 have different biomechanics, so we selected L2-5, which has a similar shape, as we thought it would be the ideal level for studying biomechanics. These lumbar vertebrae were excised by orthopedic surgeons at Khon Kaen University and fresh-frozen at -30°C. Ages at the time of death were 65, 68, 81, and 89 years. Two cadavers were male and two were female. Death was due to heart disease in two cases and old age in two cases.

First, the fresh-frozen human cadaveric lumbar spines were naturally thawed. After removing soft tissues such as muscle and fat, the screws of the Suiren PS and rod system (KISCO Co., Hyōgo, Japan) were inserted into the test specimen before mounting in the test machine to prevent changes in spinal alignment and set position after the test specimen had been mounted in the test machine. Insertion of the PS was performed by an experienced spine surgeon at an angle similar to that used in normal surgery. The screws used had a diameter of 6.25 mm and a length of 40 mm, and the rods had a diameter of 5 mm. The material of screws and rods used was Ti-6Al-4V, and the key mechanical parameters, by JIS (Japanese Industrial Standards) standards, are as follows: tensile strength: 980 MPa; yield strength: 920 MPa; elongation: 14%; and reduction of area: 20%. Also, the same screws and rods were used in all specimens.

**Figure 1:**
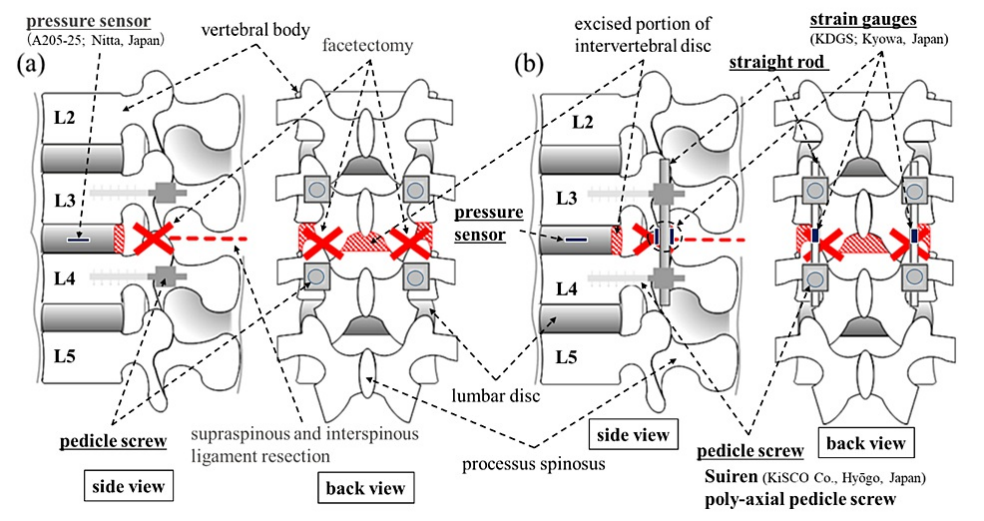
Schematic illustrations of test models (a) Control model. (b) PS-fixed model. Excision sites are shown as crosses (facet joints) and dashed lines (supraspinous and interspinous ligaments). The excised portion of the intervertebral disc is shown as the red-shaded area. PS: pedicle screw

Next, assuming an injured spine with the instability that occurs during PLF, we prepared an injury model by excising the posterior portion of the intervertebral disks to a depth of about 5 mm using Luer forceps after the complete excision of bilateral facet joints and the supraspinous and interspinous ligaments between L3 and L4. We used this injury model as a control model (Fig. 1a), considering that, when creating such an injury model, the spinal alignment would be altered and biomechanical values such as intradiscal pressure would greatly change when compared with the intact model [7]. Then, we attached the rods to the pre-placed PS in each of the L3 and L4 vertebral arches, being careful not to apply any mechanical load. This was used as the PS-fixed model (Fig. 1b). Axial compressive load tests were performed on these two models to measure the strain generated in the rod and the intradiscal pressure. Then, from the obtained data, we calculated the stress generated in the rod and the load sharing between the spine and the implant. Based on these results, we aimed to obtain basic information that can contribute to the examination of surgical techniques and the construction of numerical simulations for posterior reconstruction between single vertebrae, which is considered the most basic, as a preliminary step to examining anterior reconstruction.

Measuring device, method, and formula

**Figure 2:**
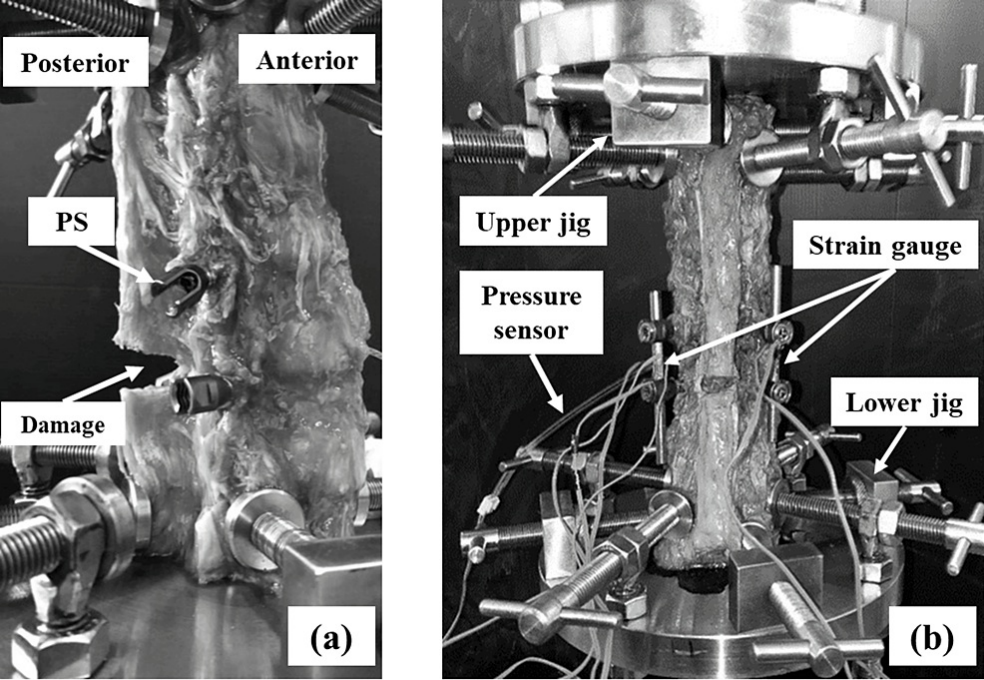
Fixation of a human cadaveric lumbar spine to the tester (a) Control model from the sagittal plane. (b) PS model from the posterior side. (a) shows the sagittal plane of the control model in which PS are inserted and the spine is set in the test machine. The supraspinous and interspinous ligaments and facet joints have been removed as damage. Looking at the anterior side of the specimen, the specimen is seen to be in lordosis. (b) shows the PS model from the posterior side. The strain gauge cords can be seen from the centers of the rods and the pressure sensor in the back on the left side. PS: pedicle screws

The ElectroPuls E10000 (Instron, Grove City, Pennsylvania, United States) universal testing machine was used for the experiments, and the cadaveric lumbar vertebrae were fixed to the testing machine using a special fixing jig (Fig. 2). As for the clamping procedure, first, the lower clamp was temporarily fixed so that the upper and lower ends (L2, L5) of the cadaveric lumbar vertebrae were located in the center of the table of the testing machine. Next, the upper clamp of the testing machine was moved to the top of the cadaver, the upper clamp was fixed, and finally, the lower clamp was permanently fixed. Therefore, the lumbar vertebrae were fixed while maintaining their lordosis. Afterward, the initial load was set to zero, and axial compressive loading was applied at a load speed of 50 N/s until reaching 700 N, following which the load was removed. During the loading test, the lordosis of the lumbar spine alignment is increased by the initial lordosis of the lumbar spine as compression progresses. Additionally, since the spine has viscoelasticity, the displacement caused by loading may be velocity-dependent, and the experimental results may differ between the first and second loading cycles. In this study, we aimed for static loading that eliminated viscoelasticity as a basic experiment and selected a loading rate as slow as possible under the conditions of this study. Furthermore, the first time was used as a pretreatment to take into account the effects of viscoelasticity, a compressive load was applied again under the same conditions, and the rod strain and intradiscal pressure at the second maximum load were measured. The maximum load was set under the assumption that 70% of body weight (1000 N) acts on the lumbar area in a standing position [8].

The measurements of (1) compressive stress and bending stress in the rods, (2) compressive load applied to the entire PS instrument, and (3) intradiscal pressure between L3 and L4 were obtained using the following methods.

**Figure 3:**
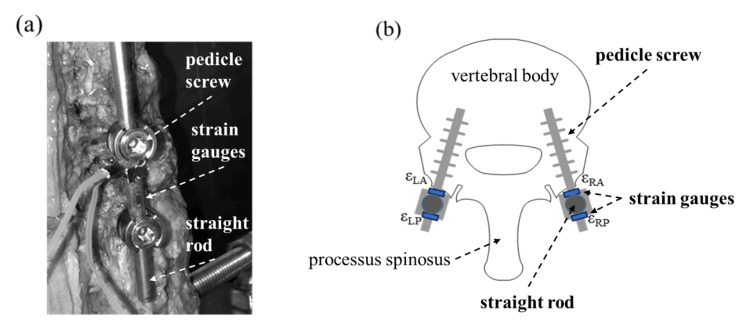
Strain gauge installation (a) The strain gauges installed on the rod are shown. (b) A schematic illustration of the strain gauges' attached position is shown. Each strain gauge is installed on the anterior and posterior sides of the rod.

First, to examine the maximum compressive strain and bending strain generated in the rods, as part of the test preparation, we affixed two strain gauges (KDGS; Kyowa Electronic Instruments Co., Tokyo, Japan) facing each other (shifted by 180 degrees in the circumferential direction) to the center of the rod's longitudinal axis (Fig. 1). Strains were measured at four locations for anterior strain (ε_LA_) and posterior strain (ε_LP_) on the left rod and anterior strain (ε_RA_) and posterior strain (ε_RP_) on the right rod (Fig. 3). Here, for the strain of the compressive element of the compound load (ε_comp_), we averaged in the anterior compressive strain (ε_A_) and the posterior tensile strain (ε_P_), and calculations were made with each strain applied to Equation 1: ε_comp_=(ε_A_+ε_P_)/2 (1).

Then, based on the relationship between stress and strain shown in Equation 2 [9], we calculated the absolute value of stress in the compressive components (σ_comp_) applied to the rods using the value of ε_comp_, σ_comp_=|Eε_comp_| (2), where E is Young's modulus for Ti-6Al-4V, the material used for the rods, defined in this experiment as E=110 GPa (1.10×1011 N/m^2^) [10].

Next, the bending strain that occurs on the rod (ε_bend_) could be obtained by subtracting the compressive strain (ε_comp_) obtained in Equation 1 from the strain values (ε_A_, ε_P_). The rods in this study were round and the cross-sectional shape showed front/back symmetry; therefore, the bending strain was the same size (absolute value) on the anterior and posterior sides of the rod. From this, bending strain (ε_bend_) was taken to be as shown in Equation 3: ε_bend_=|ε_A_-ε_comp_|=|ε_P_-ε_comp_| (3).

The bending stress (σ_bend_) applied to rods was then calculated using Equation 4, similar to the stress in the compressive components (σ_comp_): σ_bend_=Eε_bend_ (4).

Moreover, the compressive load applied to the entire PS and rod system needs to be calculated to clarify the proportion of the 700 N vertical load applied to the specimen distributed to the vertebrae and spinal column fixture. Compressive load F applied to the rods was calculated using Equation 5, and the sum of compressive load F obtained from each of the left and right rods was used as the compressive force applied to the entire PS and rod system, F=(σ_comp_)A (5), where A in this equation indicates the cross-sectional area of the rod. The rod used in this experiment has A=19.6 mm^2^ (19.6×10^-6^ m^2^).

Pressure sensors (A205-25; Nitta Corporation, Osaka, Japan) with a diameter of 9.5 mm and a thickness of 0.208 mm for the pressure-sensitive section were used to measure intradiscal pressure. After preparing the injury model, an incision was made with a scalpel in the lateral center portion of the intervertebral discs of L3 and L4, and the pressure sensor was inserted into the center of the annulus fibrosus at a depth of approximately 2 cm. The signal detected by the sensor during compressive loads was then amplified using an amplifier (amp box; Nitta Corporation, Osaka, Japan). Following A/D conversion using a PCD-320A sensor interface (Kyowa Electronic Instruments Co., Tokyo, Japan), the load applied to the pressure sensor was stored as electronic data at a sampling frequency of 10 Hz. Since the output of the pressure sensors used represented the load produced in the pressure-sensitive section, intradiscal pressure (σ) (in MPa) was calculated by dividing the load by the cross-sectional area of the pressure-sensitive section.

## Results

Table 1 shows the values of strain in the rods at a maximum load of 700 N for the PS-fixed model in the four test specimens. Compared to the results for other samples, the strain value for Cadaver 4 is about half as small. On the other hand, in all cadavers, the strain values of the rods were negative at the anterior side (compressive strain) and positive at the posterior side (tensile strain). In addition, the absolute values of those strains were confirmed to be larger on the anterior side than on the posterior side in all cadavers.

**Table 1:** Values of strain at each position of the rod at a maximum load of 700 N for the PS-fixed model of the four test specimens from strain gauges ε_LA_: strain on the anterior side of the left rod; ε_RA_: strain on the anterior side of the right rod; ε_LP_: strain on the posterior side of the left rod; ε_RP_: strain on the posterior side of the right rod; PS: pedicle screw

	Strain of load ε			
	Anterior		Posterior	
	ε_LA_(×10^-6^)	ε_RA_(×10^-6^)	ε_LP_(×10^-6^)	ε_RP_(×10^-6^)
Cadaver 1	-1040	-995	875	900
Cadaver 2	-913	-1180	853	1028
Cadaver 3	-1070	-1020	1058	923
Cadaver 4	-405	-595	390	508

Table 2 shows the results of calculating compressive and bending stresses applied to the rods using these strain values. From these results, the deformation of the spinal fixture was discovered to be heavily impacted by the bending load, with a large difference in stress levels seen between the left and right rods. In addition, the average of the total of bending moments on the rod calculated by multiplying the bending stress σbend obtained in these results by the section modulus Z=πd^3^/32=1.23×10^-8^ m^3^ of the rod (round bar, d=5 mm) was approximately 2 Nm.

**Table 2:** Result of calculation of compressive and bending stresses applied to the rods using values from Table 1

	Compressive stress σ_comp_		Bending stress σ_bend_	
	Left (MPa)	Right (MPa)	Left (MPa)	Right (MPa)
Cadaver 1	9.08	5.23	105	104
Cadaver 2	3.30	8.36	97.1	121
Cadaver 3	0.66	5.34	117	107
Cadaver 4	0.83	4.79	43.7	60.7

Table 3 shows the sum of compressive loads generated in the left and right rods at a load of 700 N, corresponding to the compressive load applied to the entire PS instrument. These results showed the compressive load applied to the PS instrument was calculated to be a maximum of 281 N, a minimum of 110 N, and an average of 184.5 N. And on average 26.3% of the 700 N load applied to each test specimen was distributed to the rods.

**Table 3:** Sums of compressive loads generated in the left and right rods at a load of 700 N

	Compressive load F		
	Left (N)	Right (N)	Total (N)
Cadaver 1	102.5	178.2	280.7
Cadaver 2	164.1	64.8	228.9
Cadaver 3	104.8	13	117.8
Cadaver 4	94	16.2	110.2

Table 4 shows the values of intradiscal pressure at a load of 700 N for each model in the four test specimens. Intradiscal pressure was discovered to be decreased when PS fixation was applied to the control model, and the rate of decrease was not very high, reaching a maximum of 40%, a minimum of 16%, and an average of 26%. Notably, the average rate of decrease in intradiscal pressure and the average compressive load applied to the PS instrument (Table 2) were approximately the same.

**Table 4:** Intradiscal pressure at a load of 700 N for each model in the four test specimens from pressure sensors PS: pedicle screw

	Intradiscal pressure σ	
	Control (MPa)	PS-fixed (MPa)
Cadaver 1	0.99	0.81
Cadaver 2	0.70	0.59
Cadaver 3	0.88	0.53
Cadaver 4	1.14	0.82

## Discussion

In this study, we conducted an axial compressive load test on human cadaveric lumbar vertebrae, targeting the most basic posterior reconstruction between single vertebrae, as a preliminary step to examining anterior reconstruction in the surgical treatment of the lumbar spine. Although it is necessary to take into account the small number of samples, we were able to obtain basic biomechanical findings that can contribute to the selection of surgical techniques for posterior fixation such as the PS system and the construction of numerical simulations for the consideration of these.

First, it is necessary to consider the fact that the rod strain results showed significantly different values for Cadaver 4; however, in all samples, it was confirmed that the absolute value of strain for both the left and right rods on the anterior side was larger than on the posterior side. Therefore, we found that even in cases when a simple axial compressive load is applied to a human cadaveric test specimen, compressive stress and bending stress on the rod are simultaneously generated due to the lordosis of the spine. In addition, the bending stress had a dominance of one or more digits over the compressive stress. In the report of Cripton et al. [5], in which posterior instrumentation was used on injured spines and a bending moment of 8 Nm was applied, the sum of bending moments produced in the rods was about 1 Nm in a model that eliminated intervertebral disc and posterior stabilizing elements and about 8 Nm in a discectomy model that eliminated intervertebral soft tissue. In an in vivo experiment by Rohlmann et al. [4], the moment acting on the rod reached a maximum of about 11 Nm from a minimum value near 0 Nm. In contrast, the average of bending moment we calculated was approximately 2 Nm. For the size of this bending moment, the present results, in addition to being in the range of the results in previous studies, showed the possibility that relatively large bending moments act on the rod even with an axial compressive load of 700 N alone. In clinical cases, many reports have described rod damage or breakage associated with long fixation, such as spinal stenosis surgery, osteotomy, and circumferential spinal osteotomy [11-13]. Based on our results, high bending stress may act on the rod even in the early postoperative period with limited lumbar flexion and other movements, in patients with such considerable instability, and consideration should be given to the design and selection of rods and screws. In addition, this research is a static mechanical test consisting of only an axial compressive load test. The bending stress calculated in the mechanical experiment was below the elastic limit of the titanium alloy, but under dynamic loading conditions such as repetition and impact, it is expected that stress two to four times higher will occur. This suggested that PS fixation alone may not be sufficient to ensure stability. Therefore, it was considered that one piece of information was obtained regarding the consideration of securing additional stability using intervertebral cages, cross-links, etc.

The present results also confirmed that the stress on the left and right rods is unequal, with large individual differences. Similar results for the variation in the asymmetry and size of the strain on the left and right rods were also reported by Rohlmann et al. [4] and Cripton et al. [5]. Asymmetry in the shape of the vertebral bodies and individual differences in the test specimens are thought to be one cause of asymmetry in the distance between screws and the angle of screw insertion. Moreover, while previous studies have shown results from bending moment load, the present results indicate that even in cases of simple axial compressive load on the spine, stress on the left and right rods is asymmetrical, and large stress is applied to one of the rods. Therefore, if one considers only the prevention of rod damage or breakage, a solid design ensuring that damage or breakage does not occur even when the load is placed on one rod only would seem to be important. For example, as stated by Jazini et al. [14], it may be necessary to make the rod diameter larger, switch to cobalt-chromium material, or make other changes to give the rods sufficient ability to withstand bending stress [15,16]. Moreover, the strain of the rod in the PS-fixed model showed (-) values on the ventral side and (+) values on the dorsal side in all test specimens. These results confirmed that the rod was deformed in a kyphotic, contrary to the lordotic, deformation behavior of the lumbar spine. We were able to confirm biomechanical findings that will help construct and verify numerical simulations to analyze spinal deformation behavior and implant stress distribution and investigate the causes of implant dislodgement and failure.

In the data on the rate of decrease in the intradiscal pressure in this study, although variation in each test specimen was confirmed, the maximum rate of decrease after PS fixation was 40%. Cripton et al. [5] reported rates of 46% in a model of posterior fixation in an uninjured spine and 38% in a model of posterior fixation in spines in which injury had been made in the intervertebral disc and posterior stabilizing elements, thus showing similar trends. Consequently, it was revealed that even in a state with only axial compressive loading, stress shielding from PS fixation is not that large, and a large load still acts on the intervertebral disc, which is an anterior element. No quantitative judgment can be made since ROM, which expresses the deformation behavior of the vertebral body, was not measured, as in the report of Cripton et al. [5]. Still, it was suggested that in the severely unstable spine of the posterior column, PS fixation alone could not adequately control the movement of the vertebral body due to axial compressive loads. Because of this, in clinical settings when the anterior stabilizing elements of the spine are judged as unstable with monosegmental intervertebral PS fixation, the provision of appropriate stabilization of the anterior stabilizing elements of the spine using a bone graft or intervertebral cage may be important to prevent bone fusion or instrument failure.

Additionally, in this study, the mean compressive load acting on the PS instrument and the mean rate of decrease in the intradiscal pressure obtained from the pressure sensor were nearly equal, at about 26%. As a result, the decrease in intradiscal pressure from PS fixation was thought to be transformed into an increase in the compressive load of the PS instrument. Because the injury model used in this study removes posterior stabilizing elements such as facet joints, it is assumed that the intervertebral disc receives most of the compressive load. Therefore, when the PS device was placed as a posterior stability element, it was considered that a compressive component of stress comparable to the reduction in intradiscal pressure was distributed to the PS device. Based on this, in assessing the risk of implant dislocation or failure due to PS-only fixation, it was considered necessary to understand the state of anterior stability, mainly the intervertebral disc. Additionally, it was assumed that a bending stress approximately 10 times greater than the compressive stress distributed to the rod, which served as the rear stabilizing element, would be generated in the rod. These fundamental findings are deemed valuable in contributing to the development and validation of effective numerical simulations for the selection of PLF and PLIF, as well as for analyzing stress distribution in screws and rods.

Limitations

The limitations that should be noted in this study were that (1) the number of test specimens was small; (2) the spinal alignment and vertebral body ROM that affect the variation in experimental results were not confirmed; (3) the only spinal implant used was PS fixation, and there was only one size; and (4) it is not possible to study the stress distribution of screws and rods. In this report, this sample size was decided because there was a limit to the number of autopsy bodies that met the exclusion criteria and could be used. Therefore, the generalizability of the results may be limited. In the future, with reference to other similar cadaver studies, we plan to increase the number of samples of cadaveric lumbar vertebrae and conduct mechanical tests, including measurements of spinal alignment and ROM, to examine the effects of different implant sizes and anterior reconstruction. Furthermore, we would like to take advantage of the information obtained from these studies and conduct studies that combine numerical simulations.

## Conclusions

Our study revealed that in cases where severe instability of the lumbar spine's posterior column is stabilized by PS fixation, even when only axial compressive load is applied, it may result in the following complex deformation behavior. The bending stress generated in the rod is more than 10 times the compressive stress and may be concentrated on one side. Rod deformation becomes kyphotic, in contrast to the lordotic deformation behavior of the lumbar spine. Stress shielding caused by PS fixation is small. Although the results of this study are foundational data, they are believed to be useful for the consideration of anterior reconstruction, constructing and verifying effective numerical simulations to investigate the causes of dislodgement and failure of spinal implants.
